# Optogenetic Therapy for Retinal Degenerative Diseases

**DOI:** 10.18502/jovr.v18i4.14542

**Published:** 2023-11-30

**Authors:** Zahra-Soheila Soheili

**Affiliations:** ^1^Department of Molecular Medicine, National Institute of Genetic Engineering and Biotechnology, Tehran, Iran

From the 1970s through the 2000s, microbial opsins were identified as a family of proteins which react to light and shuttle ions through plasma membranes. Single-celled microbes use opsins for movement toward sources of light. By optical control in the milli-second range and accuracy at the level of cell type, the optogenetic approach has unfold new horizons for understanding biology, both in states of health and disease. Optogenetic has the potential to facilitate recovery of neuronal activity in neurological disorders unrelated to mutations.

Rod and cone photoreceptors detect light and initiate visual sensation. Degeneration of photoreceptors due to a variety of causes leads to vision loss in millions of people worldwide. According to the stage of degeneration, numerous therapeutic approaches are being explored. Optogenetic therapy is an innovative and promising approach which delivers photo-activated ion channels to surviving retinal cell types including bipolar cells and retinal ganglion cells. These functional changes are aimed at restoring vision in diseases such as retinitis pigmentosa and age-related macular degeneration.^[1]^

Optogenetic performs a crucial function in neuronal specialization of stem cells through membrane depolarization. The energy transfer from light however, can cause side effects in living tissues.^[2]^ This is exemplified by the phototoxic effects of blue light, compared to red light, in optogenetic experiments. Shorter wavelength light demonstrates reduced tissue permeation as compared to red light. Scattering is the most important factor limiting light penetration within biological tissues. Shorter wavelengths of light lead to greater scatter, therefore the depth of tissue penetration of blue light is lower than that of red light.^[3,4,5]^ Light-induced toxicity can also be managed through illumination procedures. Crimson and infrared wavelengths elevate tissue temperature and induce thermal damage. A carefully planned lighting scheme and intermittent lighting can also be employed to diminish excessive tissue heating.^[7,8]^

In 2005, researchers discovered that by incorporating opsins into brain cells of rodents, the cells fired when subjected to light stimulation. By 2007, the technology achieved accurate regulation of mice behavior. Since that time, gene families of bacteriorhodopsin, halorhopsin, and channelrhodopsin have been genetically modified. The most functionally sophisticated molecules are as follows:

• ChrimsonR is an optogenetic cation channel that is optimally excited by 590 nm wavelength.

• ReaChR, red-activatable ChR, is most effectively stimulated by orange to red light spectra (λ ∼ 590–630 nm). It has improved plasma membrane trafficking and substantial photocurrent along with even quicker kinetics than red-shifted ChRs.^[9]^

• MCO (multi-characteristic opsin), functions in ambient light and a wide range of wavelengths within the visible light spectrum. Under normal lighting conditions, substantial light-induced current is the characteristic of MCO-sensitized cells with rapid kinetics necessary for eyesight. Due to sensitivity to ambient light, external light stimulation is not required for MCO-activation and therefore light-induced toxicity is not an issue. Given that MCO is a polychromatic opsin with a broad spectrum of activation, participants with MCO-sensitized retina can regain their vision in different color settings.

• eNpHR 3.0, enhanced NpHR, Natronomonas halorhodopsin enhanced for optogenetic applications, is excited with 593 nm. NpHR is a fast light-activated electrogenic Cl-pump that enables precise inhibition of distinct cells in the nervous system.

Close to two decades of optogenetic researches for vision restoration has led to multiple ongoing clinical trials in this area; (https://clinicaltrials.gov/ct2/results?cond=&term=optogenetic&cntry=&state=&city=&dist=). Even though optogenetic endow light sensitivity to neurons in the inner retina that survive following photoreceptor loss, there are major concerns with ocular optogenetic gene therapy. The specificity of delivery, control of activity, potential off-target mutations, and inherent immunogenicity are the most challenging concerns.

In one study, researchers injected AAV encoding ChrimsonR (red-light drivable Channelrhodopsins from Chlamydomonas noctigama) into the eyes of a blind patient. Via light stimulation by engineered goggles, local changes in light intensity projected light pulses onto the retina in real time. Transduced retinal ganglion cells were activated optogenetically and the patient perceived, located, counted, and touched different objects. This was the earliest described case of optogenetic gene therapy for a neurodegenerative disease leading to a functional recovery.^[10]^

We designed engineered red Opto-mGluR6 with broader, red-shifted action spectrum compared to Opto-mGluR6. ROM19, ROM18, and ROM17, as red-shifted variants of Opto-mGluR6, were designed, synthesized, and cloned into AAV-MCS-IRES-EGFP vectors. The expression of constructs was confirmed in engineered HEK-GIRK cells. Spectrophotometry and patch clamp demonstrated that they were sensitive to longer wavelengths of light and directly coupled light stimuli to G-protein signalling (unpublished data).

We also studied neuroretinal differentiation of Opto-mGluR6-engineered mouse retinal pigment epithelium (mRPE) and bone marrow mesenchymal stem cells (BMSCs) through blue light stimulation. mRPE and BMSCs were selected for optogenetic studies due to their capability to differentiate into retinal-specific neurons. mRPE cells and BMSCs were transduced by AAV-MCS-IRES-EGFP-Opto-mGluR6 viral vectors and stimulated for 5 consecutive days with blue light (470 nm). Optogenetic stimulation-induced elevated intracellular Ca ${}^{2+}$ levels in mRPE- and BMS-treated cells. Significant increase in cell growth rate and G1/S phase transition were detected in mRPE- and BMSCs-treated cultures. Expression of Rho, Thy1, OPN1MW, Recoverin, and CRABP, as retinal-specific neuron markers in mRPE and BMS cell cultures were also demonstrated.^[11]^

Ganglion cells, amacrine cells, photoreceptor cells, and bipolar and Muller precursors were determined in BMSCs-treated cultures. mRPE cells represented more abundant terminally differentiated Muller glia when compared with BMSCs. This study revealed that optical stimulation increased the intracellular Ca ${}^{2+}$ level and proliferation and differentiation of Opto-mGluR6-engineered BMSCs.^[11]^

In conclusion, optogenetic has revolutionized the field of regenerative medicine. However, there are several concerns including specificity, delivery methods, and safety. There are also challenges in realizing optogenetic potentials consisting of precision, personalized medicine, and mapping neural circuits.

## References

[B1] Daruich A, Robert MP, Bremond-Gignac D (2023). Gene therapies in pediatric ophthalmology. Front Ophthalmol (Lausanne).

[B2] Hu J, Adebali O, Adar S, Sancar A (2017). Dynamic maps of UV damage formation and repair for the human genome. Proc Natl Acad Sci USA.

[B3] Al-Juboori SI, Dondzillo A, Stubblefield EA, Felsen G, Lei TC, Klug A (2013). Light scattering properties vary across different regions of the adult mouse brain. PLoS One.

[B4] Azimipour M, Baumgartner R, Liu Y, Jacques SL, Eliceiri K, Pashaie R (2014). Extraction of optical properties and prediction of light distribution in rat brain tissue. J Biomed Opt.

[B5] Yona G, Meitav N, Kahn I, Shoham S (2016). Realistic numerical and analytical modeling of light scattering in brain tissue for optogenetic applications. eNeuro.

[B6] Ash C, Dubec M, Donne K, Bashford T (2017). Effect of wavelength and beam width on penetration in light-tissue interaction using computational methods. Lasers Med Sci.

[B7] Ziegler T, Möglich A (2015). Photoreceptor engineering. Front Mol Biosci.

[B8] Zheng B, Wang H, Pan H, Liang C, Ji W, Zhao L, et al (2017). Gong, Wu X, Chang J. Near-infrared light triggered up conversion optogenetic nanosystem for cancer therapy ACS Nano.

[B9] Wright W, Gajjeraman S, Batabyal S, Pradhan S, Bhattacharya S, Mahapatra V, et al (2017). Restoring vision in mice with retinal degeneration using multicharacteristic opsin. Neurophotonics.

[B10] Sahel JA, Boulanger-Scemama E, Pagot C, Arleo A, Galluppi F, Martel JN, et al (2021). Partial recovery of visual function in a blind patient after optogenetic therapy. Nat Med.

[B11] Shams Najafabadi H, Sadeghi M, Zibaii MI, Soheili ZS, Samiee S, Ghasemi P, et al (2021). Optogenetic control of neural differentiation in Opto-mGluR6 engineered retinal pigment epithelial cell line and mesenchymal stem cells. J Cell Biochem.

